# Conflation of prediction and causality in the TB literature

**DOI:** 10.5588/ijtldopen.25.0142

**Published:** 2025-07-09

**Authors:** M.L. Romo, L. Barcellini, M.F. Franke, P.Y. Khan

**Affiliations:** ^1^ Department of Global Health and Social Medicine, Harvard Medical School, Boston, Massachusetts, USA;; ^2^ Department of Pediatrics, "V. Buzzi" Children's Hospital, ASST FBF Sacco, Milan, Italy;; ^3^ Clinical Research Department, Faculty of Infectious and Tropical Diseases, London School of Hygiene & Tropical Medicine, London, UK;; ^4^ Interactive Research & Development (IRD) Global, Singapore, Singapore.

**Keywords:** tuberculosis, drug-resistant, epidemiologic methods, data interpretation, risk factors

## Abstract

**BACKGROUND:**

Observational data can answer both predictive and etiologic research questions; however, the model-building approach and interpretation of results differ based on the research goal (i.e., prediction versus causal inference). Conflation occurs when aspects of the methodology and/or interpretation that are unique to prediction or etiology are combined or confused, potentially leading to biased results and erroneous conclusions.

**METHODS:**

We conducted a rapid review using MEDLINE (2018–2023) of a subset of the observational TB literature: cohort studies among people with drug-resistant TB that considered HIV status an exposure of interest and reported on TB treatment outcomes. For each article, we assessed the research question, statistical approach, presentation of results, and discussion and interpretation of results.

**RESULTS:**

Among the 40 articles included, 32 (80%) had evidence of conflation. The most common specific types of conflation were recommending or proposing interventions to modify exposures in a predictive study and having a causal interpretation of predictors, with both types frequently co-occurring.

**CONCLUSION:**

Conflation between prediction and etiology was common, highlighting the importance of increasing awareness about it and its potential consequences. We propose simple steps on how TB and lung health researchers can avoid conflation, beginning with clearly defining the research question.

Studies relevant to human health can broadly be classified as descriptive, predictive, or etiologic based on the questions they aim to answer (Table 1).^1^ Descriptive studies quantify and characterize health phenomena.^2^ Predictive studies identify factors that predict an outcome or develop a prediction model.^3,4^ Etiologic studies estimate the causal effect of an exposure on an outcome to inform interventions.^5–7^ Descriptive and predictive studies typically rely on observational data.^2–4^ For etiologic studies, experimental studies with random exposure assignment (i.e., randomized trials) to address confounding are considered the gold standard, but ethical considerations as well as feasibility and practicality have driven the advancement of methods for estimating causal effects from observational data.^5^ Studies might also have simultaneous but separate descriptive, predictive, and etiologic objectives.

**Table 1.** Overarching research goals, characteristics of different components, and resources for descriptive, predictive, and etiologic studies using observational data.

	Descriptive study	Predictive study		Etiologic study
**Overarching goal conveyed in the research question or objective**	Quantify and characterize health phenomena, often a specific outcome or exposure, within a target population and time frame	Identify factors that predict an outcome being present (diagnosis) or developing over time (prognosis)	Develop a model comprising multiple factors that predicts an outcome being present (diagnosis) or developing over time (prognosis)	Estimate the causal effect of an exposure on an outcome
**Example of a research objective and potential implications of the results, with relevance to TB**	*Objective:* To describe TB preventive therapy initiation and completion rates for people with HIV on antiretroviral therapy in a public-sector HIV treatment program from 2019 through 2024 *Potential implications of results:* Gaps in TB preventive therapy implementation could inform policy and allocation of resources.	*Objective:* To identify which respiratory symptoms, spirometry measures, and/or chest x-ray findings at TB treatment completion predict subsequent post-TB lung disease *Potential implications of results:* Risk stratification to identify patients who might benefit from additional monitoring after TB treatment and possibly to inform development of a future prediction model for post-TB lung disease.	*Objective:* To develop and validate a risk score that predicts future QT prolongation risk in people initiating rifampicin-resistant TB treatment *Potential implications of results:* The risk score could be used by clinicians to inform patient monitoring strategies during treatment.	*Objective:* To estimate the causal effect of alcohol use disorders on end-of-treatment outcomes among people with drug-susceptible TB *Potential implications of results:* The findings could help determine if alcohol use disorders are an effective intervention target to improve TB treatment outcomes.
Statistical approach^*^	Descriptive measures of the outcome or exposure occurrence, such as risks or rates, are computed. Weighting or imputation may be done to increase validity. Stratum-specific or adjusted/standardized results may be included.	Variables for inclusion in the final model are identified based on their ability to predict the outcome (e.g., backward/forward selection based on statistical criteria, machine learning algorithms), rather than considering causal pathways.		Confounders are identified based on their role in the causal structure of the exposure-outcome relationship and are handled in the model using an appropriate method (e.g., multivariable adjustment, inverse probability weighting) or are considered in the analysis design (e.g., instrumental variable analysis, regression discontinuity). Tools like directed acyclic graphs may be used to understand and specify the causal structure comprising confounders, mediators, and colliders, as informed by current knowledge.
Presentation of results^*^	Main results are presented as estimates of the measures of occurrence, including overall and stratified estimates and crude/corrected estimates, as applicable.	All coefficients in the model are presented, allowing prediction of diagnosis/prognosis and risk stratification for individuals. For prediction models, relevant performance measures (e.g., area under the curve, sensitivity, calibration) are presented.		Main results are presented as relative or absolute risks in which confounding was minimized (e.g., adjusted risk differences). Coefficients of covariates (confounders) in the model are not presented.
Discussion and interpretation of results^*^	Avoids a causal interpretation of the results and overinterpreting stratum-specific differences.	Interpretation focuses on the ability of specific factors or a model with multiple factors to predict an outcome, with predictive ability based on a statistical criterion. The multivariable model is proposed for use in individuals for diagnostic or prognostic purposes, or for risk stratification. Intervening on the predictors cannot be recommended since they may not be causal of the outcome. Residual confounding is not described as a limitation since variables were selected without considering causal pathways.		Interpretation focuses on the magnitude and precision of estimates without overemphasizing statistical significance, considering possible threats to internal validity. Causal language may be used if appropriate. Actions or recommendations for interventions may be proposed based on the results. Potential underlying pathways (e.g., biological, behavioral) that explain the exposure-outcome association may be discussed. Residual confounding may be described as a potential limitation.
**Recommended resources**	A Framework for Descriptive Epidemiology^2^	Prognosis Research Strategy (PROGRESS) 2: prognostic factor research^3^ Developing clinical prediction models: a step-by-step guide^4^		Causal Inference: What If^5^ (available from: https://miguelhernan.org/whatifbook) Control of Confounding and Reporting of Results in Causal Inference Studies. Guidance for Authors from Editors of Respiratory, Sleep, and Critical Care Journals^6^

* Information in columns for predictive and etiologic studies is from the assessment criteria listed in Supplementary Data Table S2 and based on Ramspek et al.^8^ and other recommended resources.^3-6^ Information for the descriptive study column summarized from Lesko et al.^2^

The research question, model-building approaches, and interpretation of results are distinct for predictive and etiologic studies, as only the latter must consider the underlying causal structure of the exposure-outcome relationship (Table 1).^5–7^ The overarching goal of prediction is to identify who will develop (i.e., prognosis) or already has (i.e., diagnosis) an outcome, irrespective of causality. However, both predictive and etiologic studies can use the same observational data and similar statistical approaches, like multivariable regression. In this context, ‘conflation’ between prediction and etiology, i.e., combining or confusing aspects of methodology and/or interpretation, can occur.^8^ Conflation is especially relevant for observational data and is expected to be less of an issue with primary analyses of experimental studies, as randomized trials are specifically designed, analyzed, and reported to answer causal ‘what if’ types of research questions.

Conflation can potentially lead to biased results and erroneous conclusions, which may have implications for patient care and public health and the direction of a research field. If an etiologic study adjusts for variables that are solely predictive of the outcome rather than confounders (i.e., common causes of the exposure and outcome), variables on the causal pathway, such as colliders and mediators, might be included in the model and bias the effect estimate (e.g., risk difference/ratio). For example, incorrectly adjusting for the mediator of birth weight can bias the effect of maternal smoking on infant mortality towards the null.^9^ Misinterpretation of estimates from regression models in both etiologic and predictive studies can lead to erroneous conclusions. For example, estimates from predictive studies that were misinterpreted as causal contributed to the misconception of a protective effect of cigarette smoking on SARS-CoV-2 infection risk, which led to unproductive research into nicotine and possibly increased tobacco consumption during the COVID-19 pandemic.^10^

We examined conflation between prediction and etiology in the observational TB literature by conducting a review and assessment of a subset of articles on HIV and drug-resistant TB (DR-TB). Observational studies could conceivably explore HIV as a predictor or a cause of unfavorable DR-TB treatment outcomes. We assessed articles for conflation, and described the frequencies and types of conflation to provide practical recommendations for how the TB and lung health research community can avoid conflation between prediction and etiology.

## METHODS

We conducted a rapid review of the observational HIV and DR-TB literature and applied interim reporting recommendations.^11^ The review was guided by a protocol that was developed a priori by two of the authors (LB and PYK) and is summarized as follows:

Eligible articles reported the findings from cohort studies among people with DR-TB and considered HIV status as the primary factor of interest and/or included it in multivariable analyses and reported on TB treatment outcomes. We searched MEDLINE for articles in the prior five years from the search date (03 July 2023), using Medical Subject Heading and free text terms related to our topic (Supplementary Data Table S1). We excluded articles that were not in English, not a complete research report, or if the full-text article was unavailable. A single reviewer (LB) conducted the search, and title, abstract, and full-text screening. Two reviewers (LB and MLR) independently assessed study characteristics relevant to predictive and etiologic studies and evidence of conflation in four domains: research question, statistical approach, presentation of results, and discussion and interpretation of results. For this assessment, we adapted a tool with signaling questions from a prior review focused on conflation (Supplementary Data Table S2).^8^ The signaling questions probed about specific aspects unique to predictive and etiologic studies in each domain. A response of yes, no, unclear, or not applicable was recorded for each. We also included ‘Table 2 Fallacy’ whereby coefficients for non-main effects in an etiologic study (e.g., confounders) are interpreted and/or presented, typically in the second table of a research paper.^12^ Although all regression coefficients from a predictive study should be presented and interpreted, this is not the case for etiologic studies, which are typically focused on a specific exposure and interpreting coefficients of confounders may lead to incorrect conclusions about their role in the causal pathway. Based on the responses to the signaling questions, each of the four domains was classified as predictive, etiologic, conflated, or unclear. Additionally, a domain could be classified as both predictive and etiologic (without conflation) if the study clearly had separate predictive and etiologic research questions and these were treated distinctly in any given domain. The two reviewers compared their findings and came to a consensus on any discordance. Any remaining disagreements were resolved by consulting two senior researchers (PYK, MFF).

**Table 2.** Examples of selected article excerpts and assessments within each domain.

	Article excerpt^*^	Assessment
Research question	*‘The objective of this study was to compare 24-month outcomes between patients initiated on an injectable containing or bedaquiline-containing short regimen for rifampicin-resistant tuberculosis....’^13^*	*Etiologic.* Although not explicit about causality, the comparison of the effect of two different regimens on outcomes suggests a causal contrast. A predictive objective seems unlikely since the objective is focused on a single exposure (i.e., regimen).
	*‘In this study, we describe the demographics, clinical characteristics, and prognostic factors associated with treatment outcomes in these MDR-TB patients’^14^*	*Predictive.* Although the verb ‘describe’ is used, one of the underlying goals can be understood to be prediction because use of the word ‘prognostic’ in this context suggests risk stratification.
	*‘…we aimed to…determine factors that are associated with the duration from treatment initiation to death or treatment failure in children treated for DR-TB…’^15^*	*Unclear.* The words ‘factors’ and ‘associated’ are nonspecific and do not distinguish between causal and predictive factors. Not articulating a specific exposure of interest might suggest that the research question is predictive, but it might also be that the authors are interested in examining multiple causal relationships.
Statistical approach	*‘Multivariable logistic regression analysis was performed using variables found to be significantly associated or having borderline association (p-value < 0.1) with an unfavorable treatment outcome in the bivariate analysis to identify those that were independently associated with it.’^16^*	*Predictive.* Covariates were selected for the multivariable regression model based on their ability to predict the outcome (using their p-value) rather than the underlying causal structure.
	*‘We calculated unadjusted and adjusted relative risks and their confidence intervals using log-binomial regressions. The adjusted model included factors associated with the outcome at p<0.1 in binary regressions and age and sex disregarding their significance as common confounders.’^17^*	*Conflated.* As with the previous example, a data driven approach for selecting covariates is used for the multivariable model; however, age and sex are simultaneously included because of their role in the underlying causal structure (confounding). If the goal of this study was etiologic, including covariates based on a statistical criterion rather than considering their role in the causal pathway could lead to a biased effect estimate, as some of these variables may be mediators and colliders rather than confounders. If the goal of this study was predictive, including age and sex in the model without considering their predictive ability might negatively impact model performance or the precision of other estimates.
Presentation of results	*‘The odds of unfavorable outcomes remained significantly lower in the bedaquiline group after adjustment for age, CD4 cell count, HIV status, and baseline smear positivity in a multivariable logistic regression model (adjusted odds ratio [OR] 0.38; 95% CI, .18–.81).’^18^*	*Etiologic.* Causal interpretation of the main exposure of interest (bedaquiline) on the outcome (unfavorable treatment outcomes) adjusting for variables previously identified to be potential confounders. Odds ratios for the confounders are not presented, thus avoiding table 2 fallacy in the results text.
	*‘In multivariate analysis… Smoking was identified as a predictor of unfavorable outcomes in these participants (aOR 5.1, 95% CI 2.4–11.4; P<0.001).’^19^*	*Predictive.* Exposure (smoking) explicitly identified as a predictor of the outcome of interest, with results coming from a multivariable model that included multiple predictive variables.
	*‘Risk factors for unfavorable treatment outcomes, death, and loss to follow-up are shown in Tables… On adjusted analysis, unfavorable treatment outcomes were significantly higher in patients with XDR-TB, patients with increasing age, …’^20^*	*Unclear.* The term ‘risk factors’ is ambiguous. The description of outcomes being ‘significantly higher’ in certain groups cannot be interpreted as exclusively etiologic or predictive.
Discussion and interpretation of results	*‘In our study, we also identified age, history of cigarette smoking, thrombocytopenia, and anemia as significant predictors of unfavorable outcomes.’^19^* *‘…the effect of confounding variables on the outcome could not be all be controlled for.’^19^*	Conflated. The first excerpt summarizes the main findings of the study as predictive, but second excerpt describes not having data on some confounders implying that the causal structure was considered in the study design. Because of the combination of predictive and etiologic elements related to the same results in the discussion, we would classify this text as having evidence of conflation. If this were a predictive study, the statement implying residual confounding is not relevant.

* Article excerpts represent illustrative examples. Some abbreviations are written out for clarity.

Among the articles included, we computed frequencies of classifications for each domain and the article overall, and types of conflation. An article was classified as conflated if any one of the four domains was classified as conflated (i.e., containing both etiologic and predictive elements) or at least two domains had discordant classifications (e.g., the research question was predictive and the discussion and interpretation of results was etiologic). An article was classified as unclear if the research question was unclear and the criteria for conflation were not met or if the research question was both etiologic and predictive and subsequent domains were neither both nor conflated. Otherwise, an article could be classified as etiologic, predictive, or both.

## RESULTS

**Figure 1.**
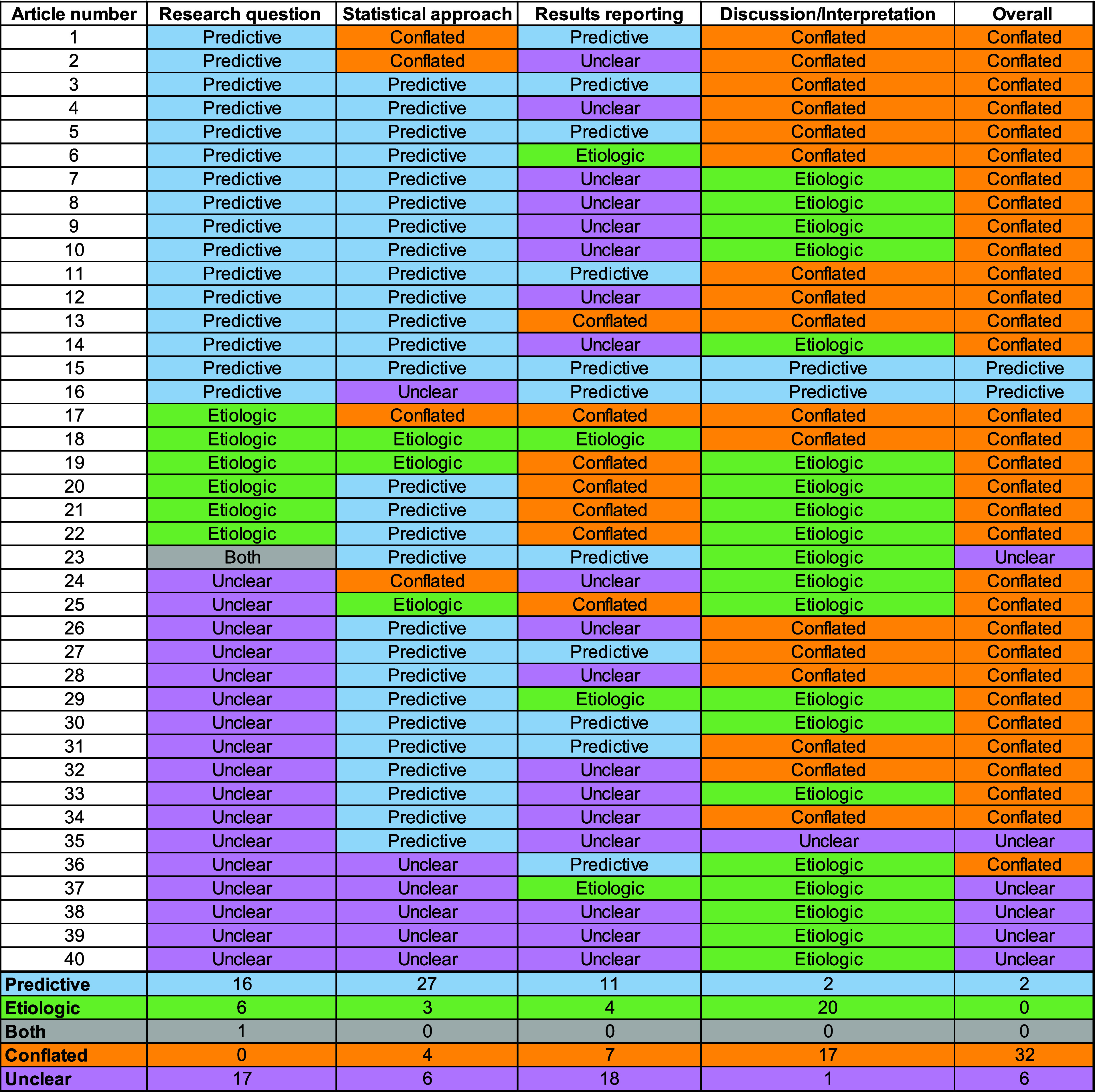
Visual representation of the assessment of domains for each publication included in the review. The top part of the figure shows how each domain was classified and the overall determination for each of the 40 articles, sorted by classification of the research question. The bottom part of the figure shows a table with summary frequencies.

Of 177 articles identified, 69 were excluded based on their title and abstract and 108 were sought for retrieval. Of these, we were unable to retrieve 7, and of the 101 full-text articles, 61 were excluded, leaving 40 articles included in our review (Supplementary Data Figure S1). Figure 1 provides classifications for the four domains and the summary classification for each article. Table 2 contains illustrative example text from selected articles from the review^13–20^ and how they were classified.

The research question domain was classified as predictive for 16 (40%), as etiologic for 6 (15%), as both predictive and etiologic for one (3%), and as unclear for 17 (43%). The statistical approach domain was classified as predictive for 27 (68%), as etiologic for 3 (8%), as unclear for 6 (15%), and as conflated for 4 (10%). The results presentation domain was classified as predictive for 11 (28%), as etiologic for 4 (10%), as unclear for 18 (45%), and as conflated for 7 (18%). The discussion and interpretation of results domain was classified as predictive for 2 (5%), as etiologic for 20 (50%), as unclear for 1 (3%), and as conflated for 17 (43%). When considering all four domains, 2 (5%) articles were classified as predictive, none as etiologic, 6 (15%) as unclear, and 32 (80%) as conflated. Of the 32 articles with evidence of conflation, 23 (72%) had at least one domain classified as conflated and the other 9 (28%) articles had discordance among the domains.

**Figure 2.**
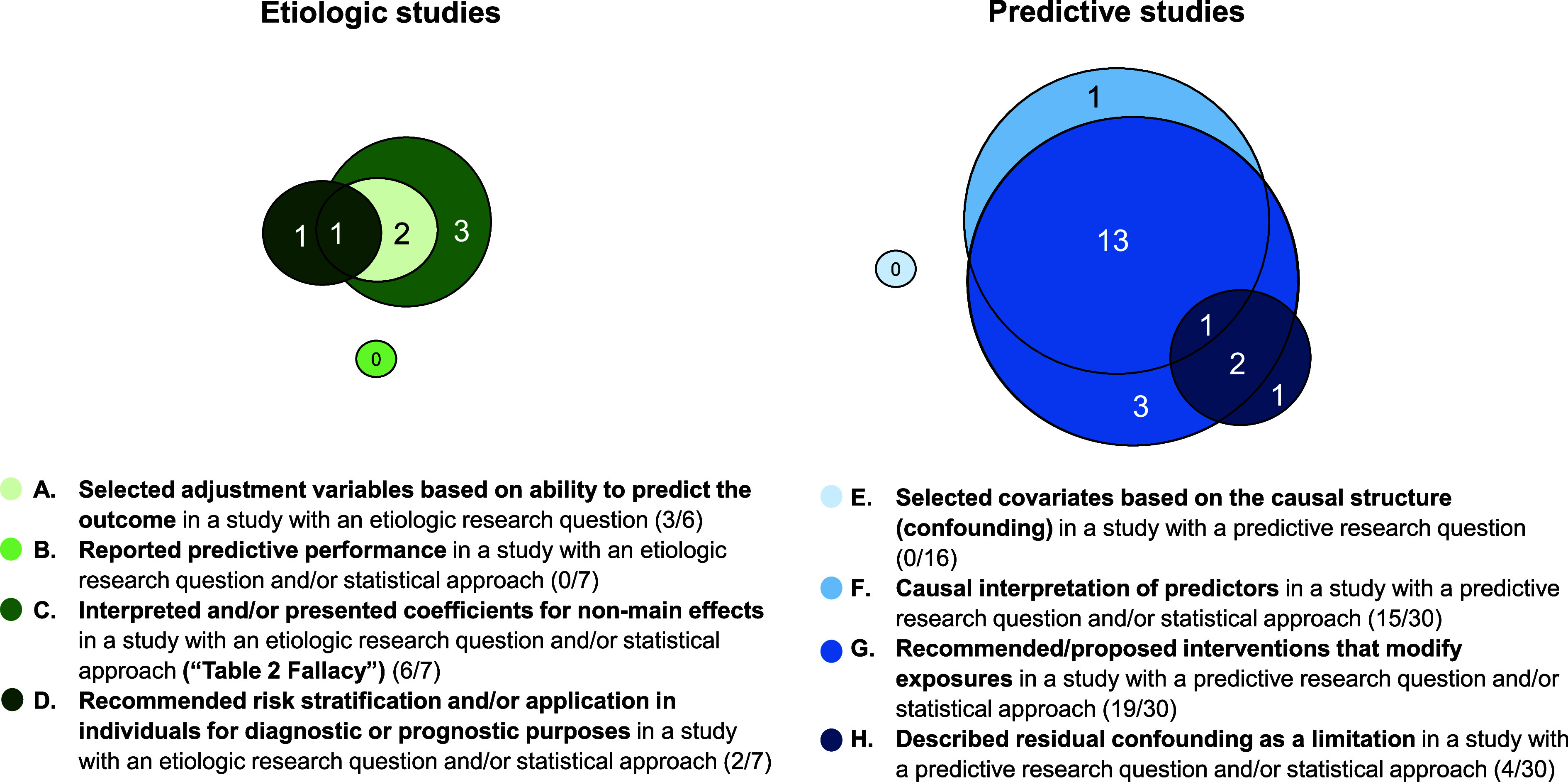
Types of conflation among etiologic and predictive studies included in the review.* ^*^Assessment of types of conflation was limited to articles that could be classified as etiologic or predictive based on their research question (types A and E) and/or statistical approach (types B, C, D, F, G, and H). One article with an unclear research question and conflated statistical approach (#24 in Figure 1) and 5 articles with both an unclear research question and statistical approach (#36-40 in Figure 1) were not included in this assessment. The one article that had both an etiologic and predictive research question and a predictive statistical approach (#23 in Figure 1) was only considered as a predictive study for the assessment of types F, G, and H. Three articles (#20-22 in Figure 1) overlapped as being classified as etiologic or predictive because they had both an etiologic research question and predictive statistical approach.

Specific types of conflation and their overlap (Figure 2) were examined for etiologic (A–D) and predictive studies (E–H). Of the 6 articles with a clearly etiologic research question, 3 (50%) had adjustment variables selected based on ability to predict the outcome (A). Of the 7 articles with an etiologic research question and/or statistical approach, none reported predictive performance (B), 6 (86%) had evidence of ‘Table 2 Fallacy’ (C), and 2 (29%) recommended risk stratification and/or application in individuals for diagnosis or prognostic purposes (D). Of the 16 articles with a clearly predictive research question, none reported selecting covariates based on the causal structure (E). Of the 30 articles with a predictive research question and/or statistical approach, 15 (50%) had a causal interpretation of predictors (F), 19 (63%) recommended or proposed interventions to modify exposures (G), and 4 (13%) described residual confounding as a limitation (H). There was substantial overlap between F and G, with 14 (93%) of 15 articles that had a causal interpretation of predictors also recommending or proposing interventions to modify exposures.

## DISCUSSION

In the recent peer-reviewed observational literature on HIV and DR-TB treatment, conflation between prediction and etiology was common. Publications with a predictive research question and/or statistical approach frequently had a causal interpretation of results and also recommended interventions to modify exposures based on the results. Additionally, we found that the specification of research questions and reporting of results were frequently unclear with respect to etiology or prediction. Compared with a previous conflation review by Ramspek et al. ours had a narrower scope (HIV and DR-TB vs. six broad medical fields) and breadth (40 articles vs. 180).^8^ They classified 127 (71%) of studies as etiologic whereas only 7 (18%) in our search had an etiologic research question and/or statistical approach. We also identified a larger proportion of articles with conflation (80% vs. 26%). These differences could be due to their search focusing on top-ranked journals, which might favor etiologic studies for their greater perceived impact and have more stringent editorial policies and rigorous peer review procedures resulting in less conflation. We agree with their recommendations for researchers and suggest the following steps to avoid conflation that are informed by our review.

### 1. Clearly define the research question(s) or objective(s) regarding description, etiology, and prediction

The research question should be clearly specified before undertaking the analysis, as analytic approaches can differ depending on the underlying goal. Effectively conveying the research question in the development of the manuscript provides the foundation for describing the statistical approach, reporting of results and the discussion, without conflating prediction and etiology. In our review, 43% of articles had an unclear research question, which was frequently related to authors stating that they examined ‘associations’ of different variables with outcomes. Since both etiologic and predictive studies examine associations between variables, clarification is needed to convey the goal of the study, which can be achieved by using more precise language. Some words that link an exposure with an outcome, like ‘cause’ or ‘prevent,’ have a less ambiguous interpretation than others.^21^ For an etiologic study, using a term like ‘causal effect’ makes the research goal clear (e.g., ‘what is the causal effect of HIV on mortality among people with rifampicin-resistant TB?’). Unfortunately, causal language is sometimes discouraged in scientific journals, particularly for observational studies,^22^ which may make it easier to conflate prediction and etiology. We encourage authors to justify their word choice if they receive opposition from reviewers and editors.

For a predictive study, authors should also use clear language about the research goal and provide context about a prognostic or diagnostic purpose, and if the goal is to identify predictive factors or develop a prediction model. For example, the question ‘what comorbidities, including HIV, predict mortality during treatment among people with rifampicin-resistant TB?’ clearly has the goal of identifying predictive factors and the description of the outcome and time horizon (i.e., mortality during treatment) implies a prognostic purpose.

### 2. Analyze the data according to the research question or objective

In the 23 studies with a clear predictive and/or etiologic research question, 8 (35%) did not have a statistical approach that matched the research question. Similar statistical methods can be used for both etiologic and predictive studies (e.g., multivariable regression). However, model-building approaches differ, with etiologic studies adjusting for confounders and predictive studies including variables that predict the outcome without regard to the underlying causal structure. Researchers should specify an appropriate analysis plan before attempting to analyze data. Such a plan should be designed for the research question, for example, by including how confounding will be addressed for an etiologic question. To ensure appropriate use of methods, we recommend engaging a methodologist whenever possible, preferably at the inception of planning a study. The review by Ramspek et al. found that conflation was less frequent when an epidemiology department was listed in the author affiliations.^8^ Individuals with training in epidemiology and/or biostatistics are ideal to engage because these fields are focused on the application of data methods for scientific inquiry.^23,24^

### 3. Report and interpret results according to the research question and methods used

The most common reasons for conflation related to reporting and interpretation of results. ‘Table 2 Fallacy’ and a causal interpretation of predictors were common among etiologic and predictive studies, respectively. A good practice to improve the quality of the reporting is to adhere to checklists like STROBE^25^ for observational studies with an etiologic objective and TRIPOD^26^ for prediction model studies, even if not explicitly required by a journal. Such checklists provide guidance to authors on what they should include in a manuscript for clearer reporting and therefore might make identifying conflation between etiology and prediction easier. However, these checklists do not recommend specific practices to avoid conflation. For example, STROBE instructs reporting unadjusted and confounder-adjusted estimates but does not explicitly state that authors should avoid reporting or interpreting coefficients from confounders for a study with an etiologic objective (i.e., ‘Table 2 Fallacy’). An important limitation is that neither TRIPOD nor STROBE is fully applicable to studies that aim to identify factors that are predictive of an outcome, which were common in our review. TRIPOD contains reporting items specific to the development, specification, and performance of prediction models, which is beyond the scope of solely identifying predictive factors. STROBE contains multiple references to confounding and confounders in both the reporting of methods and results, which are not applicable to these predictive studies.

As with defining the research question, authors must take care with the language used to interpret their findings and the conclusions they make but must also ensure that their interpretation coincides with their underlying research goal (e.g., avoid causal language when interpreting predictive associations). We found that a causal interpretation of predictors was common and frequently co-occurred with recommending or proposing interventions to modify exposures. Because factors identified as predictors might not be causal of the outcome, they could be inappropriate intervention targets. Conflating predictive results as causal might lead to recommendations or proposals for interventions targeting exposures that, even if effectively intervened upon, do not impact the outcome of interest. Many of the predictive articles in our review examined individual-level predictors of DR-TB treatment outcomes, including comorbidities such as HIV. Therefore, recommendations in the discussion should focus on risk stratification, i.e., identifying the characteristics of people at higher risk for an unfavorable outcome who might benefit from additional monitoring or support, rather than intervening on these characteristics.

Our review is subject to limitations. Some potentially eligible articles for inclusion may have been missed due to use of a rapid review methodology. However, our objective was to assess conflation between prediction and etiology within a subset of the TB literature, rather than to systematically review the evidence on the association between HIV and DR-TB treatment outcomes. Errors in interpretation may have led to some misclassification of domains and articles, but this was minimized by having two researchers independently conduct assessments, compare their findings and come to consensus, and resolve any discordances with senior researchers on the team. Furthermore, we restricted assessments to article text and avoided assumptions about the authors’ intent. Because our review focused on a subset of the TB literature, the generalizability of our findings to the broader TB literature is unknown, but we would not expect this to be an issue solely affecting articles related to HIV and DR-TB treatment. Our findings highlight the importance of increasing awareness of conflation between prediction and etiology in the broader TB and lung health research community.

Ending the world’s oldest and deadliest pandemic of TB requires leveraging the best science.^27,28^ As stated by Professor Doug Altman, ‘to maximize the benefit to society, you need to not just do research but do it well.’^29^ Achieving the best science means doing research well and reporting it well. Conflation between prediction and etiology in observational research presents an obstacle to this goal, as it can lead to biased results, erroneous conclusions, and steer a research field in an inefficient or wrong direction.^8^ We found that the avoidable phenomenon of conflation between prediction and etiology was common in a subset of the peer-reviewed TB literature, and provide practical recommendations on how to avoid it.

## Supplementary Material


