# Supplementary material for: Conflation of prediction and causality in the TB literature
Source: IJTLD Open. 2025 Jul 9;2(7):388–96. doi: 10.5588/ijtldopen.25.0142 (PMC12248412; doi:10.5588/ijtldopen.25.0142)
Supplement: Supplementary file 1 [file ijtldopen25-0142_supplementarydata1.pdf]

Table S1. MEDLINE search strategy using Ovid completed on 03 July 2023

**Observational**
**1** Epidemiologic studies/ (9351)
**2** exp case control studies/ (1427076)
**3** exp cohort studies/ (2497201)
**4** Case control.tw. (153918)
**5** (cohort adj (study or studies)).tw. (316292)
**6** Cohort analy$.tw. (11799)
**7** (Follow up adj (study or studies)).tw. (56267)
**8** (observational adj (study or studies)).tw. (161157)
**9** Longitudinal.tw. (322425)
**10** Retrospective.tw. (744498)
**11** Cross sectional.tw. (512783)
**HIV**
**12** exp “HIV infections”/ (315569)
**13** exp “HIV”/ (107523)
**14** “hiv”.tw. (350815)
**15** hiv infect*.tw. (117006)
**16** “human 1mmune?deficiency virus”.tw. (94664)
**17** “acquired 1mmune?deficiency syndrome”.tw. (16749)
**Anti-TB treatments**
**18** exp Antitubercular Agents/ (98380)
**19** ((anti-TB or anti-tubercul*) adj4 (agent* or drug*)).tw. (6356)
**MDR-TB**
**20** exp multidrug resistant tuberculosis/ or exp extensively drug resistant tuberculosis/ (10315)
**21** (multidrug resistant tuberculosis or extensive* drug resistant tuberculosis or MDR-TB or XDR-TB).ti,ab,kw. (7704)
**22** (tuberc* and (MDR or XDR or drug resistan* or multidrug resistan* or multi drug resistan* or poly drug resistan* or extensive* drug resistan*)).ti,ab,kw. (19857)
**Treatment outcomes**
**23** exp Treatment Outcome/ or exp Prognosis/ or exp Death/ or exp Mortality/ or exp Treatment Failure/ or exp Survival/ or exp Recurrence/ or exp Patient Dropouts/ or exp Patient Compliance/ (2537316)
**24** (Treatment Outcome* or Prognosis or Death or Mortality or Treatment Failure or drug treatment failure or failure or Survival or Recurrence or relapse or Patient Dropout* or dropout or non-compliance or compliance or efficacy or effective* or cure or success* or default or adheren* or conversion* or microbiologic conversion or smear conversion or culture conversion or sputum conversion).ti,ab,kw. (7734454)
**25** exp Fluoroquinolones/ or exp Quinolones/ or exp Levofloxacin/ or (fluoroquinolone* or quinolone* or levofloxacin or Levaquin or moxifloxacin or Avelox).ti,ab,kw. (74807)
**26** exp Kanamycin/ or exp Amikacin/ or exp Capreomycin/ or exp Aminoglycosides/ or (Kanamycin or Amikacin or Capreomycin or (tuberc* and injectable*)).ti,ab,kw. (182938)
**27** exp Pyrazinamide/ or exp Ethambutol/ or exp Cycloserine/ or exp Ethionamide/ or exp Prothionamide/ or (Pyrazinamide or Ethambutol or para-aminosalicylic acid or Cycloserine or Ethionamide or Prothionamide).ti,ab,kw. (16128)
**Final steps**
**28** 1 or 2 or 3 or 4 or 5 or 6 or 7 or 8 or 9 or 10 or 11 (3693765)
**29** 12 or 13 or 14 or 15 or 16 or 17 (447597)
**30** 18 or 19 or 25 or 26 or 27 (344012)

31 23 or 24 (8738045)
32 20 or 21 or 22 (22117)
33 28 and 29 and 30 and 31 and 32 (555)
34 limit 33 to (English language and humans) (501) without time limit
35 limit 34 to yr="2018 -Current**" (177) with time limit**
